# Supplementary material for: Multiplexed Host-Induced Gene Silencing of Aspergillus flavus Genes Confers Aflatoxin Resistance in Groundnut
Source: Toxins (Basel). 2023 May 5;15(5):319. doi: 10.3390/toxins15050319 (PMC10223772; doi:10.3390/toxins15050319)
Supplement: Supplementary file 1 [file toxins-15-00319-s001.zip › toxins-2316974-supplementary.pdf]

Supplementary Materials: Multiplexed Host-Induced Gene Silencing of *Aspergillus flavus* Genes Confers Aflatoxin Resistance in Groundnut

Kalyani Prasad, Kalenahalli Yogendra, Hemalatha Sanivarapu, Kanniah Rajasekaran, Jeffrey W. Cary, Kiran K. Sharma and Pooja Bhatnagar-Mathur

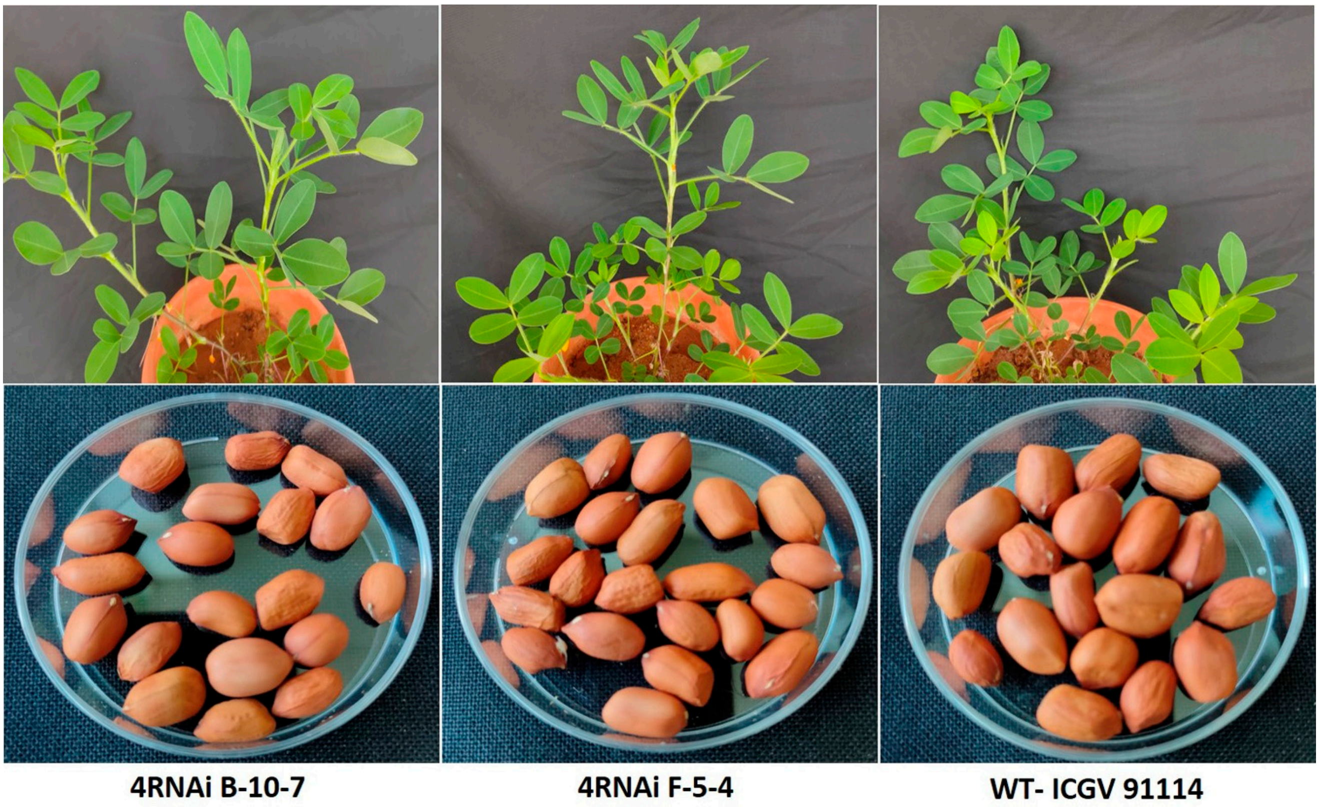

Figure S1: Phenotypic assessment of groundnut HIGS lines in T_3 generation and wild type control ICGV 91114

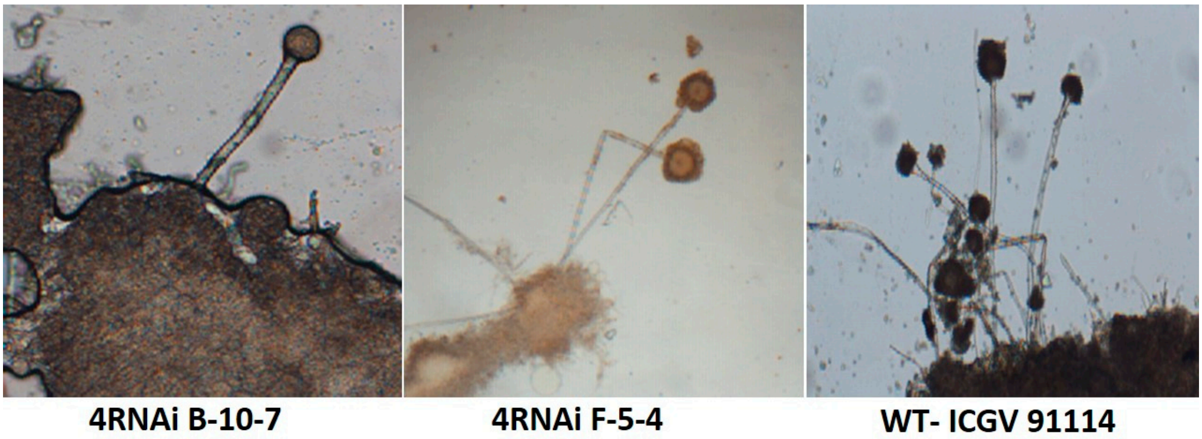

Figure S2: Microscopic observation of *A. flavus* growth inhibition in HIGS lines and susceptible wild type control

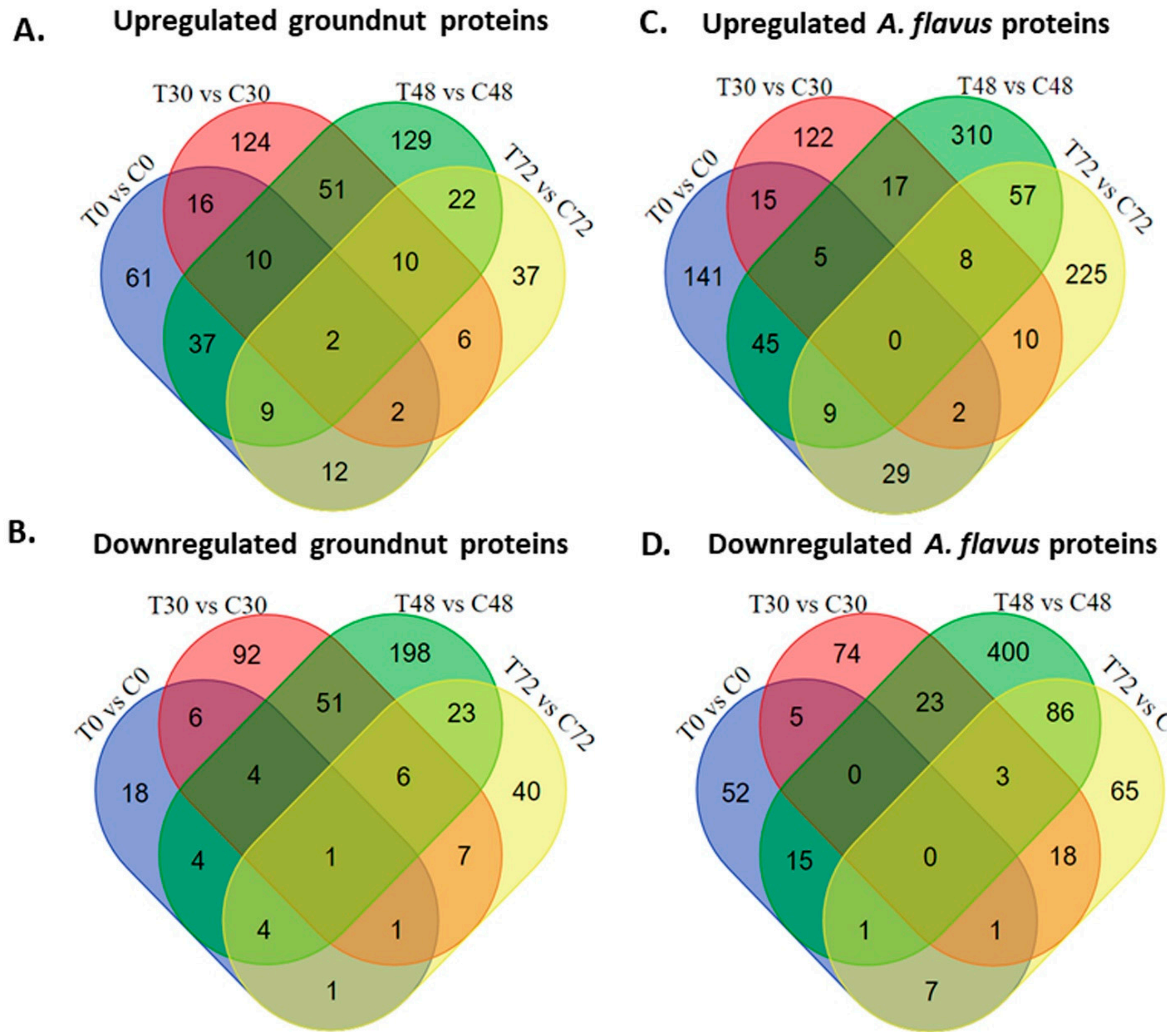

Figure S3: Venn diagram showing significantly differentially expressed groundnut and *A. flavus* proteins during infection at 0, 30, 48 and 72 hpi. (**A**) Upregulated groundnut proteins between 4RNAi and WT controls; (**B**) Downregulated groundnut proteins between 4RNAi and WT controls; (**C**) Upregulated *A. flavus* proteins between treatments 4RNAi and WT controls; (**D**) Downregulated *A. flavus* proteins between 4RNAi and WT controls.

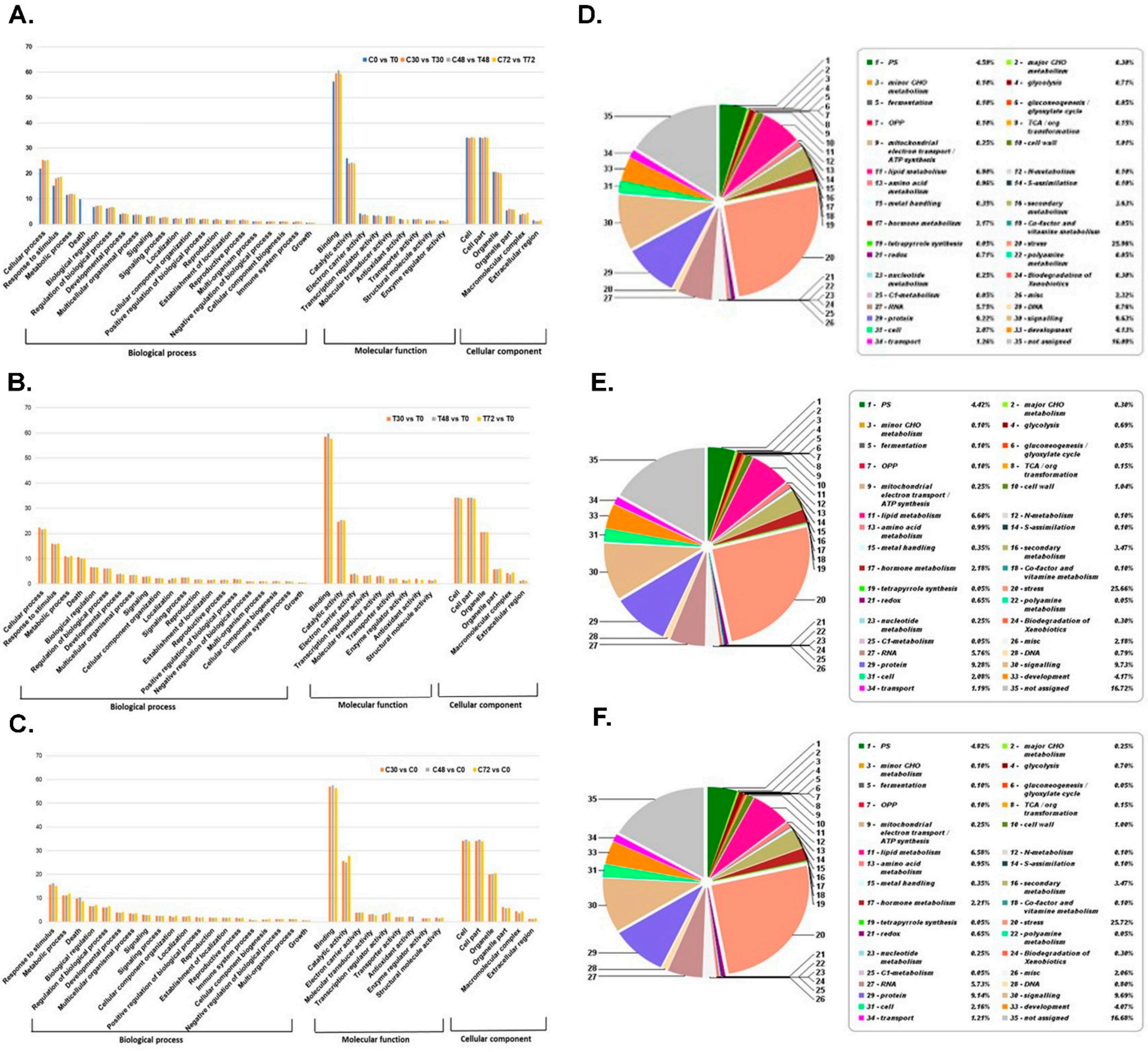

Figure S4: Protein annotation and classification. All proteins identified at different time points were classified by GO terms based on their cellular component, molecular function, and biological process in, (**A**) Between controls and HIGS events, and (**B**) HIGS events, (**C**) WT controls. D-F. Functional classification of differentially expressed proteins in (**D**) Comparison of HIGS and control samples, (**E**) HIGS, (**F**) Control, based on map man bin codes.
